# Supplementary material for: Transcriptomic Identification of Immune-Related Hubs as Candidate Predictor Biomarkers of Therapeutic Response in Psoriasis
Source: Int J Mol Sci. 2025 Aug 22;26(17):8118. doi: 10.3390/ijms26178118 (PMC12427958; doi:10.3390/ijms26178118)
Supplement: Supplementary file 1 [file ijms-26-08118-s001.zip › ijms-3699765-supplementary.pdf]

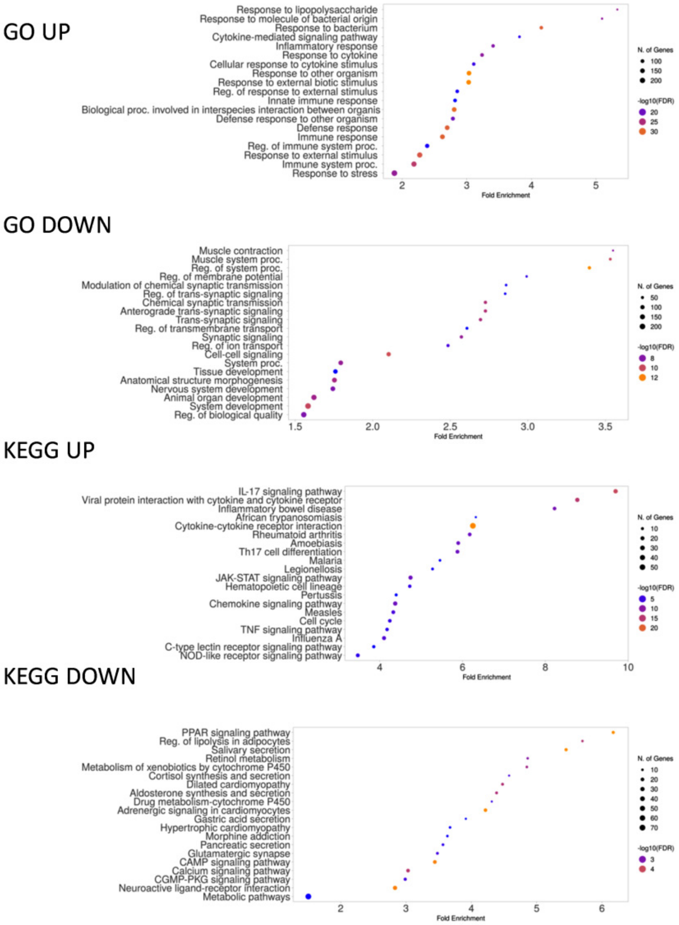

Supplementary Figure S1. Upregulated and downregulated DEGs were analyzed separately, selected by FDR, and sorted by fold enrichment.

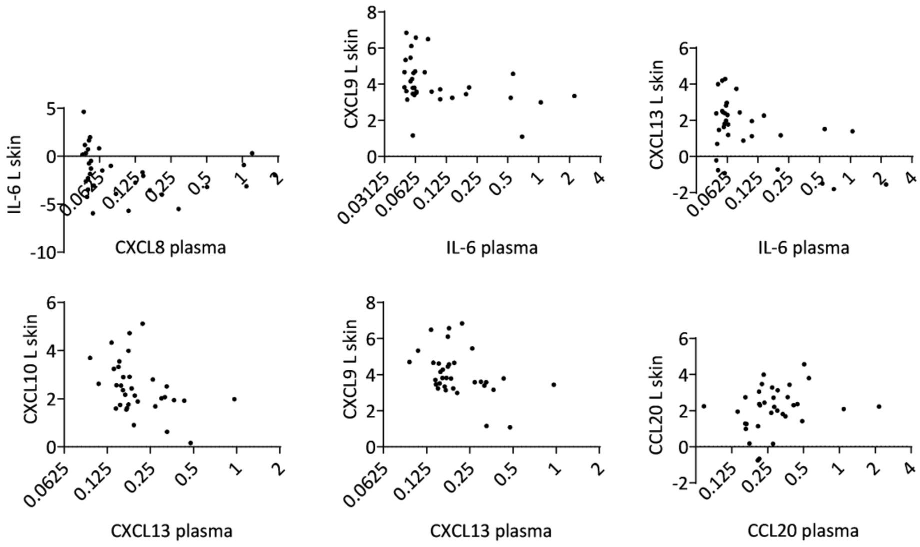

Supplementary Figure S2. Significant correlations of immune-related hubs between skin and plasma

Supplementary Table 1. Confusion matrices of each Tree predicted model

**<u>Figure 4</u>**	**Predicted: Responder**	**Predicted: Non-Responder**
**Actual: Responder**	True Positive (TP)=4	False Negative (FN)=1
**Actual: Non-Responder**	False Positive (FP)=0	True Negative (TN)=3
Accuracy: 87.5%; Sensitivity: 80%; Specificity: 80%; Precision: 100%		

**<u>Figure 5</u>**	**Predicted: Responder**	**Predicted: Non-Responder**
**Actual: Responder**	True Positive (TP)=3	False Negative (FN)=1
**Actual: Non-Responder**	False Positive (FP)=0	True Negative (TN)=7
Accuracy: 90%; Sensitivity:75%; Specificity: 100%; Precision: 100%		

**<u>Figure 7C</u>**	**Predicted: Responder**	**Predicted: Non-Responder**
**Actual: Responder**	True Positive (TP)=5	False Negative (FN)=0
**Actual: Non-Responder**	False Positive (FP)=0	True Negative (TN)=3
Accuracy: 100%; Sensitivity:100%; Specificity: 100%; Precision: 100%		

**<u>Figure 7D</u>**	**Predicted: Responder**	**Predicted: Non-Responder**
**Actual: Responder**	True Positive (TP)=4	False Negative (FN)=0
**Actual: Non-Responder**	False Positive (FP)=1	True Negative (TN)=6
Accuracy: 90%; Sensitivity:100%; Specificity: 85%; Precision: 80%		
